# Platelet Reactive Oxygen Species, Oxidised Lipid Stress, Current Perspectives, and an Update on Future Directions

**DOI:** 10.3390/cells14070500

**Published:** 2025-03-27

**Authors:** Lih T. Cheah, Matthew S. Hindle, Jawad S. Khalil, Cedric Duval, Amanda J. Unsworth, Khalid M. Naseem

**Affiliations:** 1 Discovery and Translational Science Department, Leeds Institute of Cardiovascular & Metabolic Medicine, University of Leeds, Leeds LS2 9JT, UK; l.t.cheah@leeds.ac.uk (L.T.C.); m.hindle@leedsbeckett.ac.uk (M.S.H.); j.s.khalil@leeds.ac.uk (J.S.K.); c.duval@leeds.ac.uk (C.D.); a.j.unsworth@leeds.ac.uk (A.J.U.); 2 Centre for Biomedical Science Research, School of Health, Leeds Beckett University, Leeds LS1 3HE, UK

**Keywords:** platelet ROS, oxidative lipid stress, oxLDL

## Abstract

Blood platelets are anucleate cells that play a vital role in haemostasis, innate immunity, angiogenesis, and wound healing. However, the inappropriate activation of platelets also contributes to vascular inflammation, atherogenesis, and thrombosis. Platelet activation is a highly complex receptor-mediated process that involves a multitude of signalling intermediates in which Reactive Oxygen Species (ROS) are proposed to play an important role. However, like for many cells, changes in the balance of ROS generation and/or scavenging in disease states may lead to the adoption of maladaptive platelet phenotypes. Here, we review the diverse roles of ROS in platelet function and how ROS are linked to specific platelet activation pathways. We also examine how changes in disease, particularly the plasma oxidised low-density lipoprotein (oxLDL), affect platelet ROS generation and platelet function.

## 1. Introduction

Blood platelets play a critical role in the prevention of blood loss and wound healing upon vascular injury. In circulation, the combination of shear stress and their size ensures that platelets are marginalised to the periphery of vessel where they scan for the integrity of the vessel wall. Under normal conditions, platelets are in a constant state of inhibition as a consequence of continual exposure to endothelial-derived inhibitors, prostacyclin [1], and nitric oxide [2], which prevent platelet activation through cyclic adenosine monophosphate (cAMP) and cyclic guanosine monophosphate (cGMP) signalling, respectively. Upon vascular injury, tonic inhibition of platelets is overcome to promote rapid activation and thrombus formation. The precise mechanism of platelet activation at areas of vascular damage is complex and involves a number of adhesive and soluble ligands, working through both tyrosine kinase and G-protein coupled receptor (GPCR) signalling cascades [3].

Vascular damage exposes collagen and tissue factor to the circulation, tissue factor induces the extrinsic coagulation cascade, resulting in the localised generation of the potent platelet agonist thrombin. The combination of collagen and thrombin drives platelet exocytosis of dense granules containing adenosine diphosphate (ADP), and generation of thromboxane A2 (TxA2), that act to further enhance recruitment of platelets into the growing thrombus [4]. Activated platelets also release α-granules, which translocate membrane-bound receptors to the platelet surface and assist in propagation of activation and adhesion through receptors such as P-selectin and CD40L, or soluble factors such as fibrinogen. These two receptors promote platelet-endothelial cell and platelet-leukocyte adhesion via interaction with P-selectin glycoprotein ligand 1 and CD40, enabling vascular adhesion and bridging of immune complexes to the site of injury [5].

The adhesion of platelets to the vessel wall is mediated by the interaction of previously inactive or cryptic adhesive ligands including von Willebrand factor (vWF), collagen, laminin and fibronectin [6]. Interaction between these factors and numerous glycoprotein and integrin receptors facilitates platelet accumulation at the vessel wall. Of particular interest are GPIbα, part of the larger GPIb-V-IX complex, and GPVI, which act to tether and activate platelets through vWF and collagen, respectively. Activated platelets within a thrombus differ in their morphology and function based primarily on an activation gradient [7]. Canonically, thrombi possess a sustained and potent phenotype of platelet activation in the core, with the appearance of procoagulant platelets, characterised by the redistribution of phosphatidylserine (PS) from the inner leaflet of the platelet plasma membrane to the outer leaflet [8].

In this review, we summarise the diverse roles of Reactive Oxygen Species (ROS) in platelet function and how it is linked to specific platelet activation pathways. We also examine how changes in disease, particularly hyperlipidaemia, affect ROS production and platelet function.

## 2. Reactive Oxygen Species and Platelet Activation

ROS consist of a range of oxidant species containing oxygen free radicals, such as superoxide anion (O_2_^•^), hydroxyl radical (^•^OH), peroxyl radicals (ROO^•^), and non-radical oxidants such hydrogen peroxide (H_2_O_2_), lipid peroxides (LOO^•^), and singlet oxygen (O^−^). The production of these ROS occurs during the physiological process of platelet activation. Early studies from Marcus and colleagues demonstrated the capacity of platelets to synthesise O_2_^•^ [9]. Subsequently, it was established that platelet activation led to the endogenous generation of both O_2_^•^ and H_2_O_2_, potentially ^•^OH, and that quenching of ROS diminished platelet activation [10]. Stimulation of platelets with physiological agonists, such as collagen and thrombin, produces ROS, which are required for effective platelet activation [11]. In addition, in vitro inhibition of ROS generation blocks collagen- and thrombin-mediated platelet aggregation [12]. It is widely accepted that ROS play a critical role in the signal transduction mechanisms that drive platelet activation.

### 2.1. ROS Generation in Platelets

Several sources of ROS have been identified in platelets, these include enzymatic processes such as nicotinamide adenine dinucleotide phosphate (NADPH) oxidase (NOX), cyclooxygenase-1 (COX-1), xanthine oxidase (XO), and the mitochondrial respiratory chain.

#### 2.1.1. NADPH Oxidases (NOX)

NOXs are a family of enzymes responsible for the majority of the non-mitochondrial ROS production in platelets [13]. While the NOX family is composed of seven different isoforms, including NOX1–5 and dual oxidase 1–2; to date only NOX1 and NOX2 have been shown to be expressed in both human and murine platelets [14]. Both NOX1 and NOX2 are composed of subunits that come together to form complexes upon platelet activation. The activated form of NOX1 includes the assembly of the catalytic subunit NOX1 with regulatory subunits of p22^phox^, NOX organiser 1 (NOXO1, a homologue of p47^phox^), NOX activator 1 (NOXA1), and Rac. Similarly, the activated NOX2 is composed of the catalytic subunit gp91^phox^, and the regulatory subunits of p22^phox^, p40^phox^, NOXO1, p67^phox^ (NOXA1 homologue), and Rac [15]. Activation of these enzymes is driven by protein kinase C (PKC)-mediated phosphorylation of cytosolic p47^phox^ (or its homologue NOXO1), which facilitates its interaction with membrane bound p22^phox^ and the formation of holoenzymes [16]. The binding of GTP-Rac-p67^phox^ or GTP-Rac-NOXA1 completes the active enzyme. The activated NOX complexes then transfer electrons from NADPH to molecular oxygen, producing O_2_^•^.

NOX1/2/4 triple knockout exhibit impaired platelet aggregation, adhesion, activation, and thrombus formation, and diminished O_2_^•^ production in response to collagen and thrombin [17]. These mice were protected against FeCl_3_-induced carotid artery thrombosis and collagen/epinephrine-induced pulmonary embolism; however, tail bleeding time and procoagulant PS exposure were unaffected. Elevated ROS production reduces the threshold for platelet activation and modulates endogenous inhibitory pathways [18]. Platelets from the triple knockout mice had significantly higher levels of intracellular cGMP, suggesting a link between NOX-induced ROS and platelet inhibitory signalling [17].

Many of the studies examining the role of ROS in platelets have focused on the individual NOX isoforms, which are now proposed to be activated in an agonist-specific manner and play functionally discrete roles in platelet activation, although contradictory findings have been reported (summarised in Table 1). The role of GPVI-mediated platelet activation and ROS generation is likely via NOX, although the precise NOX isoform involved remains unknown. Early studies with platelets isolated from X-linked chronic granulomatous disease (CGD) patients with genetic NOX2 (gp91^phox^) deficiency show significantly less ROS generation after collagen activation [19]. More recently GPVI-mediated activation has led to the generation of extracellular vesicles that contain NOX1, which are able to bind to and activate platelets [20]. Using NOX1- and NOX2-deficient mice, Delaney et al., demonstrated NOX1 involvement in thrombin/GPCR-induced signalling, while NOX2 was responsive to cross-linked collagen-related peptide (CRP-XL)/GPVI-induced signalling [21]. In contrast, pharmacological studies using NOX1 inhibitor ML171, combined with NOX2-deficient mice, suggest that NOX2 is not required for GPVI-mediated platelet activation [22]. To add further conflicting evidence, the simultaneous measurement of platelet aggregation with intracellular or extracellular oxygen radicals using electron paramagnetic resonance (EPR), combined with NOX-specific inhibitory peptides, suggested that NOX1 and NOX2 were linked to collagen- and thrombin-mediated activation, respectively [23].

Pharmacologically, in vivo administration of NOX1 inhibitors diminished murine platelet aggregation, oxygen radical production, thrombus formation, and carotid artery occlusion, without impacting haemostasis. NOX1 inhibition could, therefore, be a viable strategy to control collagen-induced platelet activation and reduce thrombosis without deleterious effects on haemostasis [22,23].

As with many platelet studies, the use of different pharmacological inhibitors, reliance on animal models, and the limited capacity to study long-term effects in in vitro models can limit study conclusions. Whilst discrepancies exist (summarised in Table 1) and further studies are needed to better understand the role of NOX isoforms downstream of specific agonists, it is clear that NOX enzymes play key roles in platelet activation and have subsequent targeting potential for the prevention of thrombosis.

#### 2.1.2. Cyclooxygenase-1 (COX1)

Generation of TxA2 from membrane arachidonic acid (AA) is a critical element of platelet activation. The AA liberated from membrane phospholipids, by the action of phospholipase A_2_ (PLA_2_), is metabolised through COX-1 [24]. COX is found in human platelets with 10,000 copy number per platelet [25]. The conversion of AA to the intermediate prostaglandin H_2_ leads to the generation of O_2_^•^ [26]. Platelet O_2_^•^ production by AA was almost completely suppressed in patients with inherited deficiency of gp91^phox^, the catalytic core of NOX2 [27].

#### 2.1.3. Xanthine Oxidase (XO)

XO is involved in the purine metabolic pathway, which catalyses the oxidation of hypoxanthine, a breakdown product of ATP, to xanthine, and subsequent oxidation of xanthine to uric acid producing O_2_^•^ and H_2_O_2_, respectively [28]. Studies using allopurinol, an XO inhibitor, demonstrate inhibition of platelet activation and modulation of thrombosis in dogs [29]. Although XO is not detected in the human platelet proteome studies performed by Burkhart et al. [25], XO activity ranging from 0.65 to 25.2 mU/mL was detected in platelets from healthy volunteers [30]. Additionally, increased levels of XO have been observed in platelets isolated from unstable angina patients, compared to healthy individuals, indicating that cardiac ischaemia is linked to ROS production by platelets [31]. Further studies with XO-deficient mice would be required to confirm a role in vivo.

#### 2.1.4. Mitochondrial Respiratory Chain

Mitochondria are a major source of O_2_^•^ in all cells utilising oxidative phosphorylation (OXPHOS). Mitochondrial ROS production is important for redox signalling, which links mitochondrial function to overall cell biology, but can also induce cell dysfunction in situations of mitochondrial stress. Mitochondrial ROS serve as signalling molecules in response to evolving microenvironmental changes, including coordinating the resistance and adaptation to hypoxia, activating cell survival mechanisms and regulating cellular differentiation [32]. However, excessive mitochondrial ROS production can lead to cell damage and contribute to disease pathology. Complexes I and III of the electron transport chain (ETC) are the two major loci of production of O_2_^•^ [33], which is then dismutated to H_2_O_2_ by mitochondrial manganese superoxide dismutase (MnSOD or SOD2) and subsequently to H_2_O by glutathione peroxidase (GPx). However, mitochondrial O_2_^•^ can also be released into the cytosol through the opening of the inner membrane anion channel [34], triggering elevated ROS production in neighbouring mitochondria, termed ROS-induced ROS release [35].

Platelets require high levels of ATP, and these are sourced from both glycolysis and mitochondrial aerobic respiration [36]. Several research groups have demonstrated that platelet activation by collagen and thrombin induces a rapid and transient increase in the mitochondrial membrane potential (ΔΨm) and OXPHOS, likely via Ca^2+^ mobilisation [37]. The increase in ΔΨm is associated with increased ROS generation in mitochondria, and hyperpolarisation of the membrane reduces the ETC, resulting in leakage of electrons from the chain, followed by upregulation of O_2_^•^ production [38]. The loss of ΔΨm causes the opening of the mitochondrial permeability transition pore, and release of cytochrome c from the mitochondrial matrix to the cytosol, which may play a role in apoptosis-dependent PS exposure [39].

Numerous studies have demonstrated that platelet ROS produced by the mitochondria have a significant impact on platelet function. Increased mitochondrial ROS production correlates with increased platelet activation in sickle cell disease patients, where elevated P-selectin and integrin α_IIb_β_3_ activation were attenuated with mitochondrial uncoupling and mitochondrial ROS scavenging [40]. In addition, hyperglycemia-induced ROS generation in diabetic patients was prevented in the presence of thenoyltrifluoroacetone, an inhibitor of mitochondrial ETC complex II and carbonyl cyanide m-chlorophenylhydrazone, an uncoupler of OXPHOS, suggesting that in this condition, ROS arise from the mitochondrial ETC [41]. Recently, mitoquinone, a mitochondria-targeted antioxidant, has been shown to significantly decrease mitochondrial ROS generation, subsequently inhibiting platelet expression of P-selectin and CD63 (α- and dense granule secretion, respectively), platelet aggregation induced by collagen, convulxin, PAR1, and phorbol 12-myristate 13-acetate, and platelet adhesion and spreading on collagen [42]. This new study suggests that mitochondrial ROS generation is important to many basic platelet functions.

### 2.2. Platelet Antioxidant Systems

Platelets possess a range of antioxidant systems that act to control passive and excessive ROS production, preventing potential ROS-induced platelet dysfunction. The most prominent of these endogenous antioxidant systems are superoxide dismutase (SOD), catalase, and glutathione peroxidases (GPx).

#### 2.2.1. Superoxide Dismutase (SOD)

SOD is a class of enzymes that catalyses the dismutation of the highly reactive O_2_^•^ into molecular oxygen and hydrogen peroxide. Platelets express two intracellular SOD enzymes: SOD1 and SOD2, with 13,300 and 29,500 copy numbers per platelet in human, respectively [25]. SOD1, which is found in the cytosol, is a homodimeric metalloprotein, which has a copper-zinc active site for dismutation of O_2_^•^ [43]. In contrast, SOD2, which is present in the mitochondrial matrix, has a manganese-iron active site [44], and, hence, it is also known as MnSOD. Human platelets contain approximately 1 fg of SOD/platelet, and of this approximately 77% is SOD1 [45]. Thrombus formation on collagen is significantly attenuated in the presence of SOD [46]. Several studies have assessed the potential role of SOD isoforms in regulating platelet activity in mice. Deficiency in SOD1 enhances the susceptibility to both arterial and venous thrombosis in mice, but it does not enhance platelet activation in response to thrombin, suggesting that platelet SOD1 does not confer protection against platelet hyperactivation. Instead, absence of SOD1 impairs thrombomodulin-dependent protein c activation, hence anti-coagulant activity is affected [47]. Studies using mice deficient in platelet SOD2 demonstrated increased platelet mitochondrial ROS, but overall cellular ROS was unchanged. Interestingly, platelet specific SOD2 deletion did not alter arterial thrombosis, haemostasis, or outcomes in immune disorders (sepsis and inflammatory arthritis) in which platelets play a role [48], suggesting that SOD2 is dispensable for platelet redox balance. The age of the mice used in this study was 7–15 weeks old, suggesting that low basal mitochondrial superoxide produced in platelets at young age is insufficient to cause pathologic changes even with concomitant SOD2 deficiency. During ageing, calcium elevation, mitochondrial hyperpolarisation, PS exposure, and platelet-dependent thrombin generation were exacerbated in SOD2 knockout platelets compared with control mice [49]. When a mitochondrially targeted SOD2 mimetic was applied, age-associated platelet pro-oxidant generation, procoagulant platelet formation, and in vivo arterial thrombosis were prevented [49].

#### 2.2.2. Catalase

Catalase is a tetrameric porphyrin-containing enzyme that catalyses the conversion of H_2_O_2_ to water and molecular oxygen. Catalase is present in human platelets with a copy number of 12,000 per platelet [25]. In a platelet study using rats as a model, catalase activity is detected at approximately 120 U/mg protein [50]. In human platelets, catalase inhibits collagen-stimulated TxA2 production, and release of AA from platelet membranes in a dose-dependent manner [51], corroborated by another study where collagen-induced whole blood aggregation was found to be associated with the production of H_2_O_2_, a process dose-dependently inhibited by catalase [52].

#### 2.2.3. Glutathione Peroxidases (GPx)

There are four isoforms of GPx detected in the human platelet proteome: GPx1, GPx3, GPx4, and GPx7, with the copy numbers of 34,100, 1800, 4600, and 1000 per platelet, respectively [25]. GPx are a family of selenocysteine-containing enzymes tightly coupled to the pentose phosphate pathway via reduced NADPH, which restores reduced glutathione (GSH) concentrations via GSH reductase [53]. GPx reduces hydrogen peroxide to water and lipid peroxides to their corresponding alcohols using GSH as a co-substrate [54]. GSH depletion in platelets leads to attenuated GPx activity and increased lipid peroxidation [45]. Therefore, GPx plays a crucial role in protecting cells from oxidative damage caused by lipid hydroperoxides accumulation. In platelets, GPx has been functionally linked with 12-lipoxygenase, where it oxygenates AA into 12-hydroperoxy-eicosatetraenoic acid (12-HpETE), which is then reduced into 12-hydroxy derivative (12-HETE) by a cytosolic GPx [53]. By keeping the hydroperoxides at a low level, GPx lowers the peroxide tone of platelets, preventing accelerated oxygenation of AA. It has been reported that lower GPx activity can lead to a relative accumulation of 12-HpETE [55], where such an increase may activate signal transduction pathways leading to AA release, amplifying platelet activation [56].

It has been reported that members of a family with a cerebral thrombotic disorder, exhibited reduced levels of GPx3 activity, increased circulating H_2_O_2_, and decreased NO levels in plasma [57]. Subsequent development of a GPx3-deficient mice to assess platelet function and thrombosis, demonstrated lack of GPx3 leads to enhanced ROS flux and platelet-dependent thrombosis in vivo, in part owing to the decreased NO bioavailability in the plasma [58]. GPx potentiates the inhibition of platelet function via inactivation of NO by reducing lipid hydroperoxides [59]. Suppression of NO was associated with greater platelet aggregation due to impairment of platelet inhibitory mechanisms, predisposing those affected to thrombotic complications.

### 2.3. The Role of ROS in Platelet Activation

Most ROS generation studies in platelets have focussed on activation by thrombin and collagen, although ADP [60,61] and AA [27,60] were found to also affect platelet ROS generation. Depending on the agonist, and, therefore, the stimulated receptor and signalling pathway, the ROS produced is spatially distinct within the cell [62]. Investigations using extracellular antioxidants have enabled the contribution of intracellular and extracellular platelet ROS to platelet activation to be distinguished. Stimulation of platelets with the GPVI agonist convulxin induces intraplatelet ROS production, whereas thrombin, a GPIbα and protease-activated receptor (PAR) agonist, induces mainly extracellular ROS formation [62]. There is evidence to suggest that ROS generation in response to these agonists may be linked to distinct platelet functions, and pharmacological antioxidants do not inhibit all measures of platelet activation induced by agonists. For example, a study investigated the spatial regulation of ROS, surface expression of P-selectin, and CD40L, and activated integrin α_IIb_β_3_ induced by convulxin and thrombin was found to be abolished by the GPx mimetics Ebselen and NAC, whilst the externalisation of PS was unaffected [62]. However, in the presence of both extracellular antioxidants polyethylene glycol-SOD (PEG-SOD) and catalase, only the P-selectin expression and α_IIb_β_3_ activation upon thrombin stimulation, but not convulxin, were significantly reduced [62]. Therefore, these data suggest that both intra- and extra-cellular ROS have a role in regulating the biochemical steps in platelet activation, and spatially resolved, site-specific ROS, may be targeted to control platelet activation via either thrombin or collagen. However, more studies are required to validate this.

ROS production downstream of GPVI is biphasic with the initial phase being spleen-tyrosine kinase (Syk) independent, while the second phase is Syk dependent. The initial burst of ROS occurs within 2 min of GPVI-mediated activation, followed by additional ROS production reaching a plateau after 15–20 min. In the presence of a Syk inhibitor, BAY61-3606, there was no effect on the initial ROS burst, but a complete inhibition of the second phase of ROS production [63]. These pathways may involve an interaction of GPVI with TNF adapter receptor 4 (TRAF4) [64], which acts to link PKC-δ-regulated p47^phox^ phosphorylation, translocation, and NOX2 activation, resulting in ROS generation. On the other hand, thrombin-induced ROS generation is reported to require both GPIbα- and PAR4-mediated signalling through focal adhesion kinase (FAK) and NOX1 activation [65]. This may involve cleavage of the thrombin-binding site on GPIbα, and be linked specifically to PAR4, as well as involving cyclophilin A (CyPA). CyPA has been identified as a vital component in the generation of ROS under conditions of thrombin stimulation [66]. In vascular smooth muscle cells, CyPA has been shown to interact with the p47^phox^ subunit to modulate the assembly of the cytoplasmic membrane NADPH complex, which generates ROS [67]. In CyPA-deficient mice, there is reduced thrombin-induced ROS formation and platelet activation via the integrin α_IIb_β_3_ [66].

Whilst the link between agonist stimulation and ROS generation in platelets is well established, the association between ROS and its downstream effects is relatively under-examined, although some mechanisms are known. Protein tyrosine phosphatase (PTP) is one of the proteins that is oxidised by ROS, where its active site cysteine residue, in the PTP domain, loses its catalytic activity following ROS oxidation [68]. PTPs are crucial regulators of GPVI-mediated signalling pathways in platelets [69,70]. It has been demonstrated by Jang et al. [71] that Src homology region 2 domain-containing PTP-2 (SHP-2) is oxidised by ROS generated in collagen-activated platelets, inactivating SHP-2 and promoting collagen-induced phosphorylation of downstream signalling molecules, subsequently enhancing granule secretion, α_IIb_β_3_ activation, aggregation, and thrombosis. In addition, O_2_^•^ can react with GSH, producing oxidised glutathione GSSG and more O_2_^•^ [72]. Such reaction decreases the GSH/GSSG ratio and affects the redox regulation of protein thiol groups, increasing platelet sensitivity towards agonists activation via calcium mobilisation [73]. A previous study has demonstrated that O_2_^•^ contributes to thrombus formation by disrupting the redox potential-dependent regulation (GSH/GSSG ratio) of the platelet α_IIb_β_3_ integrin [74,75], owing to the fact that the extracellular domains of α_IIb_β_3_ contain disulphide bonds, which on reduction activates the receptor and induces aggregation [76]. However, more work is needed to further our understanding of the redox-dependent changes in the biochemistry of the signalling proteins involved in platelet activation.

### 2.4. ROS and Antioxidants in Disease and Ageing

The imbalance between ROS generation and antioxidant mechanisms leads to increased oxidative stress, which is a common pathophysiological mechanism associated with various cardiovascular diseases (CVD) and platelet dysfunction.

Changes in antioxidant protection has been associated with platelet hyperactivity in several disease states including unstable angina and myocardial infarction [31], type 1 and 2 diabetes (T1D, T2D) [77], coronary artery stenosis [78], and autoimmune thrombocytopenia (ITP) [79]. Basal platelet thromboxane B2 (TxB2), the stable catabolite of TxA2, has been shown to be higher in T1D and T2D patients compared to controls, whereas platelet malondialdehyde (MDA) level, which assesses the overall lipid peroxide level, was only higher in T2D platelets, but cytosolic GPx activities were lower in platelets from both patient groups [77]. These data indicate that increased ROS and impaired antioxidant capacity may contribute to the increased risk of thrombotic occurrence of vascular diseases, and particularly in T2D patients. In murine models of streptozotocin-induced T1D, GSH levels, and the expression of antioxidant enzymes GPx-1 and SOD1, were lowered in platelets [80]. Treatment with the ROS scavenger n-acetylcysteine (NAC), a precursor of GSH, restored the antioxidant reserve of the platelets and protected T1D mice against the risk for stroke. However, a contradictory study has reported that the activities of SOD and catalase in platelets from T2D patients showed no difference in comparison with healthy controls [81]. In coronary artery stenosis patients, reduced mean platelet GPx activity and increased MDA production have been reported, which could be a contributing factor for the development of coronary artery disease [78]. Alongside this, in patients with unstable angina and myocardial infarction, a remarkable increase in platelet XO activity and MDA levels, with concomitant reductions in the activities of SOD, catalase and GPx, was found, clearly indicating the critical role of oxidants and antioxidants in ischaemic heart conditions [31]. Interestingly, a study of platelets from ITP patients, in the active phase of disease, demonstrated increased platelet oxidative stress, reduced antioxidant capacity, and increased platelet activity, which returned to normal during remission [79]. These data suggests that oxidative stress and platelet activation are implicated in the active phase of ITP.

In addition to disease states, ageing (40–79 years old) has also been shown to be associated with increased platelet ROS, decreased antioxidant activity (catalase, SOD), and enhanced activation (sCD62p, sCD40L) [82]. Intriguingly, beyond 79 years old, there is an improved platelet phenotype and reduction in hyperactivity, due to enhanced levels of platelet antioxidant enzymes and improved redox homeostasis [82], suggesting an age-associated adaptive mechanism. However, it is important to note that, in this oldest age group (>79 years old), the elevation in activated integrin α_IIb_β_3_ levels was the highest in comparison to other age groups [82], indicating that this element of platelet activation is not correlated to the antioxidant status.

## 3. Platelet Reactive Oxygen Species and Hyperlipidaemia

The inflammatory milieu associated with atherothrombosis shifts platelet towards a maladaptive phenotype characterised by hyperactivity, and this is associated with a systemic pro-thrombotic phenotype and increased ROS [83,84]. Patients with T2D and coronary artery disease exhibit increased platelet reactivity, and have increased prospective risk for coronary events and death [85]. Hyperlipidaemia, a key driver of atherothrombosis, causes a change in platelet biology in which aberrant ROS production plays a critical role. The appearance of pathological ligands in the blood, such as oxidised lipids, acting as endogenous damage associated molecular patterns (DAMPs), interact with pattern recognition scavenger receptors including CD36, scavenger receptor A1, and lectin-like oxLDL receptor-1 (LOX-1), and promote unregulated platelet activity exacerbated by ROS.

### 3.1. oxLDL and Platelet ROS

**Figure 1:**
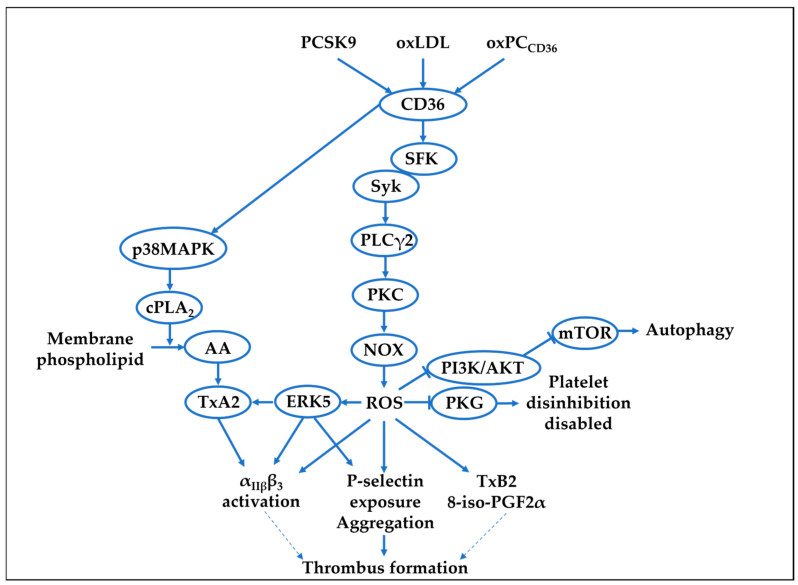
Signalling mechanisms involved in hyperlipidaemic-induced ROS production 399 and the downstream effects [23,79,80,81,82,83]. 8-iso-PGF2α, 8-iso-prostaglandin F2α; AA, arachidonic acid; cPLA_2_, cytosolic phospholipase A_2;_ ERK5, extracellular signal-regulated kinase 5; mTOR, mechanistic target of rapamycin; NOX, NADPH oxidase; oxLDL, oxidised low-density lipoprotein; oxPC_CD36_, oxidised phospholipid specific to CD36; p38MAPK, p38 mitogen-activated protein kinases; PCSK9, proprotein convertase subtilisin/kexin 9; PI3K/AKT, phosphoinositide-3-kinase/protein kinase B; PKC, protein kinase c; PKG, protein kinase G; PLCγ2, phospholipase C gamma 2; ROS, reactive oxygen species; SFK, Src family kinases; Syk, spleen-associated tyrosine kinase; TXA2, thromboxane A2; TXB2, thromboxane B2.

Hyperlipidaemia, which is characterised by significantly elevated plasma lipids, including oxidised low-density lipoprotein (oxLDL), a circulating DAMP known to activate human and murine platelets [86,87,88], increases platelet–endothelial interactions [89]. Platelets recognise oxLDL via the cell surface receptors CD36 and LOX-1, which lead to ROS generation. Table 2 and Figure 1 summarise the mechanisms of platelet ROS generation in response to hyperlipidaemia and its downstream platelet functions.

OxLDL propagates platelet aggregation and enhances platelet-dependent thrombosis, and these effects have been shown to be diminished by peptides targeting oxLDL receptors CD36 or LOX1, acting through NOX2 inhibition [90]. Our work, and that of others, has dissected the downstream signalling mechanisms of oxLDL-CD36 ligation leading to ROS production (Figure 1). A report by Assinger et al., identified a role for oxLDL-CD36 in the induction of ROS production alongside calcium flux, platelet activation, and expression of CD40L, and this activation was shown to be sensitive to the non-specific NOX inhibitor apocynin, suggesting ROS production may be via NOX1, NOX2, or both [95]. OxLDL treatment of platelets also leads to increased autophagy, a highly conserved pathway mediated by lysosomes that degrades cytosolic components, by modulating the PI3K/AKT/mTOR signalling pathway (Figure 1), which was reversed by the ROS scavenger NAC, suggesting that ROS acts as a critical signalling node in this pathway [91]. Our group has identified NOX2 as the primary ROS-generating system in platelets linked to hyperlipidaemia, which influences both activatory and inhibitory pathways. Ligation of CD36 by oxLDL led to PLCγ2-mediated ROS production (Figure 1). This ROS production was required for P-selectin expression, which was ablated by genetic deletion of CD36 or PLCγ2, inhibition of NOX2, but not NOX1, and the ROS scavenger NAC [92]. As NOX1 has been previously implicated in thrombin-mediated signalling, and NOX2 in collagen-mediated signalling, the observation that NOX2 is critical to oxLDL-ROS is supported, as collagen-GPVI signals via a tyrosine phosphorylation cascade, which shares many similarities with the signalling of oxLDL-CD36.

In addition to driving platelet activation, there is strong evidence that the CD36-NOX2 pathway can influence procoagulant function in platelets. OxLDL causes the production of catalase-sensitive H_2_O_2_, which in turn activates the MAP kinase ERK5, which leads to platelet activation and increased PS exposure required for the binding of coagulation factors [93]. Furthermore, our group found that NOX2-dependent ROS generation is driven by oxLDL-CD36-PKC signalling (Figure 1), and this promotes platelet activation through inhibition of the inhibitory NO-cGMP signalling pathway, preventing downstream activation of the protein kinase G (PKG) substrate vasodilator-stimulated phosphoprotein (VASP) [18]. Phosphorylation of VASP by PKG at serine 239 has been demonstrated to inhibit cytoskeletal rearrangement and prevent platelet integrin activation [96]. Platelets from hyperlipidaemic apolipoproteinE-deficient (ApoE^−/−^) mice have reduced sensitivity to cGMP when tested ex vivo [18]. When these mice were infused with the NOX2-inhibitor peptide gp91 ds-tat for 4 weeks, platelet hyposensitivity to cGMP was corrected, showing that platelet NOX2 may be a target for controlling platelet hyperactivity under hyperlipidaemic conditions (Table 2). Overall, current data indicate that ROS downstream of oxLDL-CD36 drives platelet degranulation and procoagulant function and disinhibits key inhibitory pathways (Figure 1). The kinetics of ROS production by oxLDL sustained over 3 h (as longest time point tested) [92] are in stark difference to those induced by haemostatic agonist such as a collagen and thrombin, which peak rapidly and then wane. It is attractive to speculate that as oxLDL concentration increases in the circulation, it drives maladaptive platelet functions through chronic and low-grade ROS production, eventually overcoming intrinsic antioxidant capacity leading to a persistent hyperactive platelet phenotype.

### 3.2. PCSK9 and Platelet ROS

Another DAMP, proprotein convertase subtilisin/kexin type 9 (PCSK9), a serine protease mainly synthesised by the liver [97], is also elevated in hyperlipidaemia, and it has been shown to have direct effects on platelet ROS production. There is evidence showing that plasma PCSK9 levels are associated with future risk of CVD events [98]. In healthy volunteers, PCSK9 binds to CD36 in platelets, activates Src, ERK5, and JNK, enhancing ROS production and further activating the p38MAPK/cPLA_2_/COX-1 signalling downstream of CD36 [94]. Aspirin, which is a COX1 inhibitor and attenuates ROS-enhanced platelet activation [99], abolishes the effects of PCSK9 on platelet activation and in vivo thrombosis [94]. Similar effects were found in atrial fibrillation patients where the investigators observed that PCSK9 levels over 1.2 ng/mL exhibit higher H_2_O_2_ production, urinary 8-iso-PGF2α biosynthesis, and serum sNOX2-dp, compared to those lesser than 1.2 ng/mL PCSK9 [100]. This study also reported that PCSK9 forms an immune complex with CD36 and initiates the subsequent cascade, including ROS generation by NOX2 activation (Figure 1).

### 3.3. Mitochondrial ROS and Hyperlipidaemia

As previously mentioned, mitochondrial ROS play a key role in many aspects of cell signalling [101]; however, its role in platelets activated by oxLDL has not yet been fully explored. Chatterjee et al. have demonstrated that oxLDL triggers intracellular rise in ROS and intraplatelet mitochondrial O_2_^•^production [102]. Interestingly, both oxidative conversion and intraplatelet lipid peroxidation were significantly reduced by MnTMPyP, a cell-permeable SOD2-mimetic O_2_^•^scavenger, suggesting that mitochondrial O_2_^•^could drive lipid peroxidation in platelets. OxLDL-induced α_IIb_β_3_ integrin activation and P-selectin expression were also shown to be inhibited by MnTMPyP. Furthermore, thrombogenic PS exposure and mitochondrial membrane depolarisation were observed, corroborating the mitochondrial O_2_^•^ generation observations. Other than platelets, endothelial cells treated with oxLDL show an increase in mitochondrial O_2_^•^, and a functional shift in mitochondrial phenotype towards impaired enzyme activity in mitochondrial respiratory chain complexes [103]. Studies of oxLDL-treated macrophages in vitro and hyperlipidaemic diet-induced macrophages in vivo identified a significant shift towards an increased rate of oxidative phosphorylation and elevated mitochondrial ROS, which is dependent on CD36 [104]. There is also evidence linking LPS-stimulated LOX1-mediated ROS production to mitochondrial function in macrophages [105]; therefore, it is possible to hypothesise that oxLDL-LOX1 interactions may lead to similar outcomes in platelets. Whether platelets also show a shift in mitochondrial function and bioenergetics in the context of hyperlipidaemia is yet to be explored; however, as the techniques required for these studies are now established in the field of platelet biology [36], these research questions should be pursued. In summary, mitochondrial dysfunction underpinned by excess O_2_^•^ and hyperpolarisation may drive changes in platelet function in various diseases, which are typically associated with a hyperactive platelet phenotype.

## 4. Conclusions and Future Perspectives

There is an ever-expanding field of research into the role of disease-mediated ROS in platelet hyperactivity, which is underpinned by molecular studies identifying both specific activators of ROS production and molecular generators of ROS, followed by pre-clinical translational studies. While multiple mechanisms have been suggested to mediate the dysfunction of platelet ROS in diseases, what remains consistently clear is that the failure of ROS homeostasis within various diseases is central to platelet hyperactivity. In many of these cases, targeting the activators that drive ROS production is unfeasible, and in such situations the development and application of ROS scavengers may provide an alternative approach, which can blunt platelet hyperactivity without losing platelet function. For instance, the antioxidant NAC, a synthetic derivative of L-cysteine, is typically safe and well tolerated at high doses in a range of CVD clinical trials [106,107,108]. Clinical studies have shown the effectiveness of NAC in improving cardiovascular functions, including reduced infarct size [107] and preserved left ventricular function in MI patients [106,108], yet its effect in platelet functions was underexamined. In vitro, NAC inhibits platelet function, aggregation, adhesion to collagen matrix, ROS generation, and intracellular calcium mobilisation [109]. In addition, the low bioavailability of NAC [110,111], due to poor membrane permeability [112], could potentially limit its clinical potential. N-acetylcysteine amide (AD4/NACA) with improved lipophilicity and hydrophobicity as well as thioredoxin mimetic peptides offer anti-aggregatory activity induced by collagen [113]. Therefore, these peptides, which display higher redox potency over NAC, could be tested in clinical settings as potential antioxidant approaches for treatment of CVD against platelet hyperactivity.

## Figures and Tables

**Table 1:** Discrepancies in the roles of NOX downstream GPCR and GPVI signalling pathways.

Agonists	Mice/ Inhibitor	Observations		Conclusions	**Ref.**
CRP (0.5 µg/mL) Thrombin (0.018 or 0.025 U/mL)	NOX1^−/−^	ROS Aggregation ATP secretion Ca^2+^ mobilisation	Thrombin ↓ CRP ↔	NOX1 is involved in GPCR-induced platelet activation	[21]
	NOX2^−/−^	ROS Aggregation ATP secretion Ca^2+^ mobilisation	Thrombin partial CRP ↓	NOX2 is involved in GPCR- and GPVI-induced platelet activation	
	NOX1^−/−^ and NOX2^−/−^	Carotid artery occlusion Tail bleeding	NOX1^−/−^ ↔ NOX2^−/−^ ↓ NOX1^−/−^ ↔ NOX2^−/−^ ↔	NOX2 is involved in thrombosis NOX1 and NOX2 are dispensable for haemostasis	
Collagen (3 µg/mL) Thrombin (0.1 U/mL)	NOX1^−/−^	Aggregation	Thrombin ↔ Collagen ↓	NOX1 is involved in GPVI-induced platelet activation	[23]
	NOX2^−/−^	Aggregation	Thrombin ↓ Collagen ↔	NOX2 is involved in GPCR-induced platelet activation	
	NOX1^−/−^ and NOX2^−/−^	Carotid artery occlusion Tail bleeding	NOX1^−/−^ ↓ NOX2^−/−^ ↔ NOX1^−/−^ ↔ NOX2^−/−^ ↔	NOX1 is involved in thrombosis NOX1 and NOX2 are dispensable for haemostasis	
	2-APT (NOX1 inhibitor)	Superoxide anion Aggregation Static adhesion over collagen Thrombus formation under flow Carotid artery occlusion Tail bleeding	Thrombin ↓ Collagen ↓ Thrombin marginally↓ Collagen ↓ ↓ ↓ ↓ ↔	NOX1 inhibition impairs GPVI-induced platelet activation without affecting GPCR responses	
Collagen (10 µg/mL) CRP (5 µg/mL) Thrombin (0.25U or 1U/mL)	NOX1/2/4^−/−^	Superoxide anion Aggregation α_IIb_β_3_ P-selectin PS exposure Carotid artery occlusion Tail bleeding	Thrombin ↓ Collagen /CRP ↓ Thrombin ↔ CRP ↓ ↓ ↔	NOXs are critical for GPVI-induced platelet activation. GPCR-associated integrin activation and platelet aggregation are NOX-dependent, whilst P-selectin and PS exposure are NOXs independent	[17]
	NOX1^−/−^	Aggregation Thrombus formation over collagen	Thrombin ↔ Collagen ↓ ↔	NOX1 is involved in GPVI- and GPCR-induced platelet activation	
	NOX2^−/−^	Aggregation Thrombus formation over collagen	Thrombin ↓ Collagen ↔ ↓	NOX2 is involved in GPCR-induced platelet activation	
	NOX4^−/−^	Aggregation Thrombus formation over collagen	Thrombin ↔ Collagen ↔ ↔	NOX4 has negligible role in platelet regulation	

↓, decreased upon agonist stimulation; ↔, unaffected; 2-APT, 2-Acetylphenothiazine; ATP, adenosine triphosphate; CRP, collagen-related protein; GPCR, G protein-coupled receptor; GPVI, glycoprotein VI; NOX, NADPH oxidase, PS, phosphatidylserine; ROS, reactive oxygen species.

**Table 2:** Effect of ROS in platelet hyperlipidaemia.

Hyperlipidaemic Insult	Platelet Function Downstream of ROS Production	ROS Related Inhibitors Used	Pathway Involved	**Ref.**
oxLDL	α_IIb_β_3_ activation, TxB2 production, thrombosis under shear stress	-Gp91ds-tat	CD36/LOX1-p38MAPK/PKC-NOX2	[90]
oxLDL	aggregation, P-selectin, adhesion under shear stress	NAC	PI3K-AKT-mTOR	[91]
oxPC_CD36_	P-selectin	NAC Gp91ds-tat ML171	CD36-Src-PLCγ2-NOX2	[92]
oxLDL	aggregation	VAS2870 DPTA-NONOate PEG-catalase	CD36-Src-NOX-ERK5	[93]
oxLDL oxPC_CD36_	diminished sensitivity to inhibitory NO-cGMP signalling	TEMPOL MnTMPyP Gp91ds-tat	CD36-Src-Syk-PLCγ2-PKC-NOX2	[18]
PCSK9	thrombin-induced platelet aggregation	VAS2870	CD36-Src-ERK5-JNK-ROS-p38MAPK/cPLA_2_/COX-1/TxA2	[94]

AKT, protein kinase B; cGMP, cyclic guanosine monophosphate; COX-1, cyclooxygenase-1; cPLA_2_, cytosolic phospholipase A_2_; DPTA-NONOate, dipropylenetriamine diazeniumdiolate; ERK5, extracellular signal-regulated kinase 5; Gp91ds-tat, peptide inhibitor for NOX; JNK, c-Jun N-terminal kinases; LOX1, lectin-like oxLDL receptor-1; ML171, 2-acetylphenothiazine; MnTMPyP, superoxide dismutase mimetic; NAC, N-acetylcysteine; NO, nitric oxide; NOX, NADPH oxidase, oxLDL, oxidised low-density lipoprotein; oxPC_CD36_, oxidised phospholipid specific to CD36; p38MAPK, p38 mitogen-activated protein kinases; PCSK9, proprotein convertase subtilisin/kexin 9; PEG-catalase, polyethylene glycol-catalase; PI3K, phosphoinositide-3-kinase; PKC, protein kinase C; PLCγ2, phospholipase C gamma 2; mTOR, mechanistic target of rapamycin; ROS, reactive oxygen species; Src, proto-oncogene tyrosine-protein kinase; Syk, spleen-associated tyrosine kinase; TEMPOL, 4-hydroxy-2,2,6,6-tetramethylpiperidine-1-oxyl; TxA2, thromboxane A2; TxB2, thromboxane B2; VAS2870, NOX inhibitor.

## Data Availability

Not applicable.
